# Bone metastases in pheochromocytomas and paragangliomas: a narrative review of presentation, diagnosis and management

**DOI:** 10.3389/fendo.2025.1671486

**Published:** 2025-11-05

**Authors:** Jorge Eduardo Contreras-Saldarriaga, Melissa Loaiza-Alzate

**Affiliations:** ^1^ Department of Endocrinology, University of Antioquia, Medellín, Colombia; ^2^ Fundación Universitaria San Martín - Sede Medellín, Sabaneta, Colombia

**Keywords:** pheochromocytoma, paraganglioma, bone metastases, neuroendocrine tumor, skeletal-related event (SRE)

## Abstract

**Background:**

Bone metastases (BM) are a frequent and clinically relevant manifestation in patients with metastatic pheochromocytomas and paragangliomas (mPPGL).

**Objective:**

This narrative review aims to summarize the current understanding of the pathophysiology, epidemiology, clinical presentation, complications, quality of life impact, and management of BM in mPPGL.

**Summary:**

Bone and lymph nodes are among the most common metastatic sites in malignant PPGL. Skeletal involvement—particularly in paragangliomas—is associated with a high incidence of skeletal-related events (SREs), including pathological fractures, spinal cord compression, and pain. These complications lead to reduced mobility, loss of independence, and decreased survival. Advances in functional and anatomical imaging have improved detection, but optimal management remains complex and requires a multidisciplinary approach.

**Conclusion:**

Recognizing patients at higher risk and understanding the biological mechanisms underlying bone dissemination are essential to optimize diagnosis, prevent SREs, and improve outcomes in this rare and challenging disease.

## 1 Introduction

Pheochromocytomas (PHEOs) and paragangliomas (PGLs) (collectively, PPGLs), are rare neuroendocrine tumors (NETs) derived from chromaffin cells of the adrenal medulla (PHEOs) and extra-adrenal paraganglia (paragangliomas) (1, 2). All PPGLs are considered to possess metastatic potential, with metastases occurring in approximately 10-15% of PHEOs and 15-40% of PGLs (3, 4).

Bone metastases (BM) are frequently identified in patients with solid malignancies, with lung, prostate, and breast cancers being the most common sources of skeletal involvement (5). In metastatic pheochromocytomas and paragangliomas (mPPGL), bone is also one of the principal sites of metastasis, with reported prevalence varying across studies (6, 7).

BM in patients with PPGLs are a significant clinical concern, leading to Skeletal-related Events (SREs) that diminish both quality of life (QoL) and survival. Complications include pain, fractures, neurological issues like spinal cord compression, rare hypercalcemia, and interventions such as surgery or radiation therapy (6). These patients also endure reduced mobility, loss of autonomy, impaired QoL, and lower overall survival (OS) rates (7), highlighting the impact of BM on patient outcomes. Early diagnosis and timely intervention are crucial to reducing the risk of morbidity and complications associated with BM (7).

Data derived primarily from case reports, retrospective studies, and, less frequently, clinical trials have demonstrated the efficacy of various treatments for managing mPPGL. However, treatment efficacy at specific anatomical sites, such as bone, has not been extensively addressed due to the rarity and heterogeneity of these tumors (6).

BM represent thereby a significant clinical challenge in patients with PPGL. Taking all the above into consideration, this narrative review aims to provide a comprehensive overview of BM in PPGL. It outlines the general characteristics, clinical presentation, and complications of these lesions, and discusses current diagnostic and therapeutic approaches. Key challenges in management and areas requiring further research are also highlighted.

## 2 Materials and methods

This narrative review was based on a comprehensive literature search conducted in PubMed/MEDLINE and Embase databases, with Web of Science additionally consulted to cross-check and confirm the bibliographic completeness of the retrieved references. The search covered publications from database inception through July 15, 2025, and the last search was performed on July 15, 2025, prior to manuscript submission.

Only articles published in English were included, encompassing original studies, case series, reviews, and clinical guidelines. Additional references were identified through manual screening of the bibliographies of retrieved articles and relevant reviews, to ensure inclusion of studies potentially missed by the electronic search.

A summary of the detailed search strings for each database, the Boolean term combinations, and the approximate number of records screened is provided in **Supplementary Appendix 1, Table 1**.

Given the narrative design of this review, no formal systematic review process or quantitative synthesis was performed.

## 3 Epidemiology

Population-based studies confirm that PPGL are rare, with incidence rates ranging from 1.9 to 6.6 cases per million person-years across regions. Reported estimates include 3.7–5.7 per million in the Netherlands (8), 6.6 per million in Canada (9), and up to 6.6 per million in Denmark (10), while a global meta-analysis reported 1.9 per million person-years (11). Regarding prevalence, a recent meta-analysis including only patients with PHEO reported a global pooled estimate of 19.8 cases per 1,000,000 individuals (≈2 per 100,000; 95% CI: 9.6–40.8) (11). In contrast, a Danish nationwide study encompassing both PHEO and PGL found a point prevalence of 64.4 per million inhabitants as of 2015 (10). Importantly, PPGLs display a strong genetic background, with germline mutations identified in approximately 26–41% of patients across contemporary mixed PPGL cohorts (12–15), particularly in younger patients (16, 17), those with bilateral or multifocal tumors (18, 19), and in extra-adrenal disease (17, 19, 20). The most frequently involved genes include Succinate Dehydrogenase Complex Subunit B (SDHB), Succinate Dehydrogenase Complex Subunit D (SDHD), Von Hippel-Lindau (VHL), RET, Succinate Dehydrogenase Complex Subunit A (SDHA), Succinate Dehydrogenase Complex Subunit C (SDHC), Succinate Dehydrogenase Complex Subunit AF 2 (SDHAF2), Neurofibromatosis type 1 (NF1), TMEM127, and MAX also contributing but at lower frequence (12, 15, 21, 22). Germline SDHB mutations confer the highest metastatic risk in PPGL, with metastatic rates ranging from 12–41% across cohorts (21) and an adjusted odds ratio of 5.68 versus non-SDHB genotypes (23). Cohort data confirm a ~29% metastatic rate among SDHB carriers (24). In a Saudi cohort, 28.6% of SDHB carriers developed metastases, markedly exceeding rates observed in other genes (22), further confirming SDHB as the predominant driver of metastatic behavior in PPGL (25). Metastasic risk varies among non-SDHB genes: SDHD ≈4%, SDHA ≈16%, SDHC ≈23%, and SDHAF2 none (21); VHL and MEN2A show moderate risk (26),NF1, TNEM127, MAX, FH and EPAS1 rarely metastatic (27). These data highlight the importance of genetic testing for all PPGL patients, as specific mutations, particularly in SDHB, are closely linked to metastatic potential, including bone dissemination (21, 28–33).

**Figure 1:**
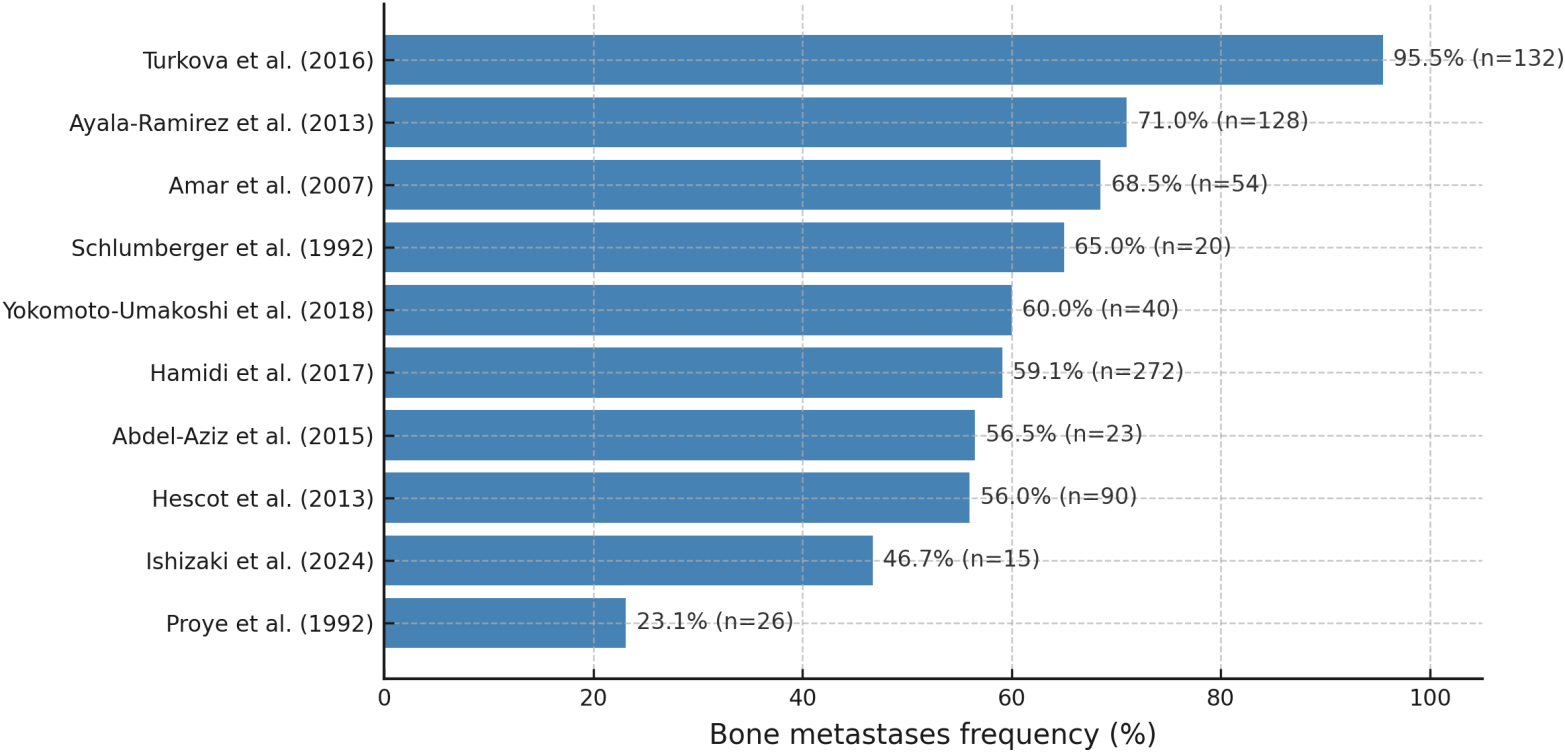
Frequency of BM in patients with mPPGL, by study.

Bone is one of the primary sites of metastasis in patients with PPGLs (7, 34–36), and in up to 20% of cases, the skeleton represents the sole site of metastatic spread (7). mPPGLs are among the solid tumors with a particularly high tropism for bone, with involvement rates comparable to those observed in other osteotropic malignancies such as breast cancer (6, 7, 37). However, data from reported cohorts indicate that the prevalence of BM is highly variable and influenced by differences in study populations. The epidemiological findings across studies are summarized in **Figure 1** and in **Table S2 (Supplementary Appendix)**. Direct evidence shows that germline SDHB and SDHA mutations confer predisposition to BM in PPGL. SDHB mutations involve bone in up to 80% of metastatic cases (38), while SDHA mutations also show marked bone tropism when metastases occur (14, 39).

Further insights into the epidemiology of BM in PPGLs have been provided by retrospective institutional series. At MD Anderson Cancer Center, a study involving 128 patients reported that the median time from primary tumor diagnosis to the development of BM was 3.4 years (7). In the same study, the presence of bone-limited disease was associated with a significantly longer median OS (12 years) compared to patients with both osseous and non-osseous metastatic involvement (5 years) (7).

A retrospective review from the National Hospital Organization Kyoto Medical Center, including 40 patients with mPPGL, identified a significant association between extraskeletal extension of spinal lesions and the occurrence of SREs (37). In this same study, the median time from the diagnosis of BM and the onset of the first SRE was 11.9 months (37).

In a larger cohort of 294 patients with bone-metastatic PPGL, 31% developed SREs, with a median time of 4.4 months (range: 0–246.6) after BM diagnosis. Among these patients, 22% underwent surgical treatment and 46% received radiotherapy (36).

Additionally, a multi-institutional study that included 100 patients with PHEO or sympathetic PGL among a broader NET population reported BM in 25% of cases (40). However, it is important to note that this estimate was not limited to individuals with confirmed metastatic disease, and therefore may not be directly comparable to studies focused exclusively on mPPGL cohorts.

Regarding the primary anatomical locations of PPGLs that give rise to BM it has been reported that these lesions can originate from both adrenal and extra-adrenal sites. In a recent large multicenter cohort of 294 patients with PPGL-related BM (36), the most common primary tumor sites were adrenal PHEOs (41%) and abdominal or retroperitoneal PGLs (30%), followed by head and neck PGLs (14%), with pelvic and thoracic locations being less frequent (5% each). Interestingly, BM have also been reported to arise from uncommon sites, such as PGLs located in the sellar region (41). These findings highlight the anatomical diversity of PPGLs associated with skeletal dissemination.

## 4 Pathophysiology

Due to their rarity, the pathophysiology of PPGL-related BM remains poorly understood; however, it is likely to reflect mechanisms observed in other malignancies with skeletal involvement. Bone is considered a preferential site for metastasis due to the extensive vascularization of the bone marrow. Tumor cells can express adhesion molecules that facilitate their attachment to marrow stromal cells, and the subsequent release of growth factors supports tumor cell proliferation and expansion within the bone microenvironment (42).

In the specific context of PPGL, it has been hypothesized that tumor cells strongly express the chemokine receptors CXCR4 and CCR9, while bone marrow stromal cells and osteoblasts secrete their corresponding ligands, CXCL12 and CCL25, respectively. This ligand-receptor interaction may facilitate the recruitment and homing of mPPGL cells to bone tissue (7, 42, 43).

Once established in the bone, tumor-induced skeletal damage may result from both catecholamine-dependent and catecholamine-independent mechanisms. Among the latter, tumor cells may disrupt the physiological balance between bone resorption and formation by promoting osteoclast activation and bone matrix degradation. This results in the development of lytic or mixed lesions, potentially exacerbated by the secretion of tumor-derived mediators that alter the bone microenvironment and favor metastatic progression (44).

Regarding catecholamine-mediated mechanisms, these hormones are known to play a significant role in bone metabolism by inducing imbalanced remodeling through activation of β-adrenergic receptors (2). In patients with PPGL, catecholamine excess may lead to increased bone turnover, with bone resorption surpassing formation (45). One study suggests that catecholamine excess and sympathetic overstimulation in PHEO can impair trabecular bone microarchitecture, reduce bone mass, and increase bone resorption, thus positioning PHEO as a potential secondary cause of osteoporosis (46). Indirect evidence further supports this hypothesis, as studies have shown that individuals treated with beta-blockers (a class of drugs that inhibit β-adrenergic signaling) exhibit a lower risk of fractures and improved bone microarchitecture compared to non-users (47, 48). Additional insights from neuroendocrine neoplasms in general suggest that bone metastases arise from a “vicious cycle,” in which tumor cells stimulate osteoclast activation primarily through RANKL overexpression and suppression of osteoprotegerin, leading to excessive bone resorption, release of growth factors from the bone matrix, and further tumor proliferation within a hypoxic, acidic, and calcium-rich microenvironment (49).

## 5 Clinical presentation and complications

BM in PPGLs, although variably prevalent, can result in significant clinical manifestations and complications that substantially impair patients’ QoL (7, 34–36). BM most commonly affect the vertebrae, spine, pelvis, long bones, ribs, and skull (7, 50), and are predominantly osteolytic (92%) and multiple (88%) in nature (37). These lesions compromise skeletal integrity and predispose patients to a range of debilitating complications, including severe pain, pathological fractures, spinal cord compression, hypercalcemia, and other SREs, all of which negatively impact QoL and functional status (7, 36, 42, 51). Notably, it has been reported that in 31% of patients with SREs, BM was the initial clinical manifestation that led to the diagnosis of malignant PPGL, underscoring that in a substantial proportion of cases, skeletal involvement can precede recognition of the underlying tumor (7).

PPGLs may also contribute to secondary osteoporosis, potentially increasing the risk of fragility fractures, particularly of the vertebrae (52). Notably, extraskeletal invasion of the spine has been identified as a significant risk factor for SREs, such as vertebral fractures and spinal cord compression (37). In one study, extraskeletal spinal invasion was significantly associated with the development of SREs (p = 0.001), suggesting that this radiological finding may serve as a valuable clinical risk marker (37).

Skeletal manifestations in this population can be severe and may require urgent multidisciplinary evaluation by neurosurgical and/or orthopedic teams, especially in cases involving spinal instability or neurological compromise (7).

Evaluation of bone health (including bone mineral density (BMD) and trabecular bone score [TBS]) is important in patients with PPGL, as both parameters have been shown to improve following surgical resection of the tumor (2, 52). Additionally, mild bone marrow suppression is a frequent finding in patients with BM from malignant PPGLs (53).

The clinical outcomes of skeletal involvement in PPGL have been described in several cohorts, whose findings are summarized in **Table 1** to illustrate the frequency and nature of bone-related complications (7, 36, 37). SREs were common but showed variability across studies. In the study by Ayala-Ramirez et al. (7), 72% of evaluable patients experienced at least one true SRE, with severe pain, pathological fractures, and spinal cord compression occurring in 33%, 27%, and 25% of cases, respectively. In contrast, Laganà et al. (36) reported a lower overall frequency of SREs (31%) in a larger cohort (n = 294), with bone pain (49%), pathological fractures (19%), and spinal cord compression (16%) being the most common manifestations. Hypercalcemia was rare in both studies. Similar outcomes were observed in a Japanese cohort of 24 patients with BM, where 50% developed at least one SRE (Yokomoto-Umakoshi et al., 2018 (37)). In this group, the most frequent SRE was radiation to bone (42%), followed by spinal cord compression (21%), severe bone pain (33%), pathological fracture (8%), and bone surgery (8%). No cases of hypercalcemia were reported, and the median time to the first SRE was 11.9 months. These findings, though derived from a smaller cohort, are consistent with the heterogeneity observed in the larger studies and highlight the need for prospective evaluation of skeletal complications in mPPGL.

**Table 1:** Frequency of SREs in three large cohorts of patients with bone-metastatic PPGL.

Skeletal-Related Event	Ayala-Ramirez et al., 2013 (7)	Yokomoto-Umakoshi et al., 2018 (37)	Laganà et al., 2024 (36)
Inclusion criteria	91 patients with PPGL and BM; 67 evaluable for SREs	24 patients with PPGL and BM	294 patients with PPGL and BM
≥1 SRE (true SREs only)	48/67 (71.6%)	12/24 (50%)	90/294 (30.6%)
≥2 SREs	23/67 (34.3%)	7/24 (29.2%)	22/294 (7.5%)
Pathological fracture	13/67 (19%)	2/24 (8.3%)	55/294 (18.7%)
Spinal cord compression	12/67 (17.9%)	5/24 (20.8%)	47/294 (16.0%)
Hypercalcemia	1/67 (1.5%)	0/24 (0%)	11/294 (3.7%)
Severe bone pain	16/67 (23.8%)	8/24 (33.3%)	144/294 (49%)*
Surgery for bone lesions	12/67 (17.9%)	2/24 (8.3%)	64/294 (21.8%)
Radiotherapy for bone lesions	31/67 (46.2%)	10/24 (41.7%)	136/294 (46.3%)
Time to first SRE	4.3 months	11.9 months	4.4 months

*Pain in Lagana et al. Was reported as a clinical symptom, not as a formal SRE.

A multi-institutional study that included 100 patients with PHEO or sympathetic PGL within a broader NET cohort reported that, in the PPGL subgroup, 20% developed spinal cord compression—consistent with previous reports. Additionally, pathological fractures and hypercalcemia were observed in 8% and 12% of PPGL patients with BM, respectively (40).

Metastatic manifestations of PPGLs can be classified as synchronous, occurring concurrently with the initial diagnosis, a scenario less frequently observed but associated with poorer prognosis (36, 54). Alternatively, they may present as metachronous metastases, which develop subsequent to the primary diagnosis and represent the more prevalent pattern (36, 55). In the study by Ayala-Ramirez et al., the median time to the first SRE was 4.4 months. This time frame is comparable to that observed in other cancers that often metastasize to the bone, such as breast cancer (56). In the MD Anderson Cancer Center study, 38% of patients presented with synchronous BM, while 63% developed metachronous BM, with a median onset of 3.4 years after the diagnosis of the primary tumor (7). Similarly, in the cohort reported by Laganà et al. (2024), 33% of patients had synchronous and 67% metachronous BM, with a longer median latency of 5.7 years (range, 0–48 years), underscoring the need for long-term surveillance in patients with PPGL (36).

Metachronous cases illustrate that skeletal metastases can occur even decades after initial diagnosis (57, 58), with intervals of up to 46 years (59) and even 52 years after the initial diagnosis in a reported case of PGL (51). This underscores the need for long-term follow-up in patients with PPGLs. The mechanisms driving the prolonged dormancy of certain metastatic PGL remain unclear. Bone-residing metastatic cells can stay quiescent for extended periods, ranging from months to decades. Reactivation of these dormant cells is hypothesized to result from alterations in chromatin structure and shifts in the tumor microenvironment (60).

**Figure 2:**
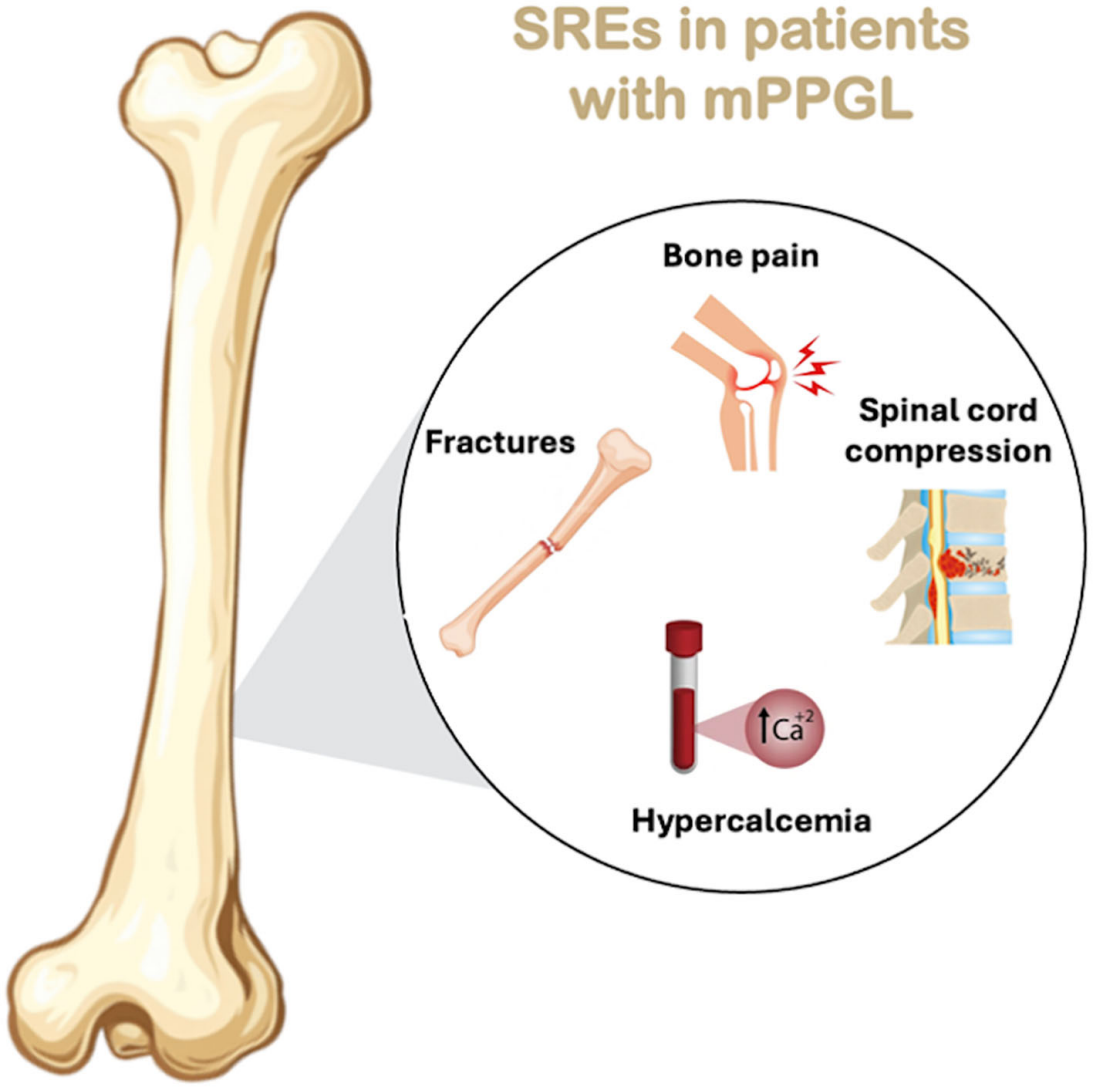
SREs in patients with mPPGL.

In the study from MD Anderson Cancer Center, SREs often occurred after BM diagnosis and, in some cases, were the first sign of malignancy (7). See **Figure 2** which graphically shows the SREs in patients with mPPGL.

## 6 Risk factors of SREs

Risk factors for SREs in patients with mPPGL have been evaluated in both single-center and multicenter cohorts, revealing complementary insights. In a smaller Japanese series, Yokomoto-Umakoshi et al. (37) identified extraskeletal invasion of the spine as a strong predictor of SREs, with affected patients showing a significantly higher likelihood of developing one or more SREs compared to those without this radiological feature (73% vs. 0%, p = 0.001). This anatomical marker of local aggressiveness also correlated with the occurrence of multiple SREs during follow-up (75% vs. 7%, p = 0.002), while no associations were found with clinical variables such as age, tumor size, functional type, or extent of metastases. In contrast, the large multicenter study by Laganà et al. (36) identified the presence of liver metastases—a marker of systemic disease aggressiveness—as an independent risk factor for SREs in multivariable analysis (p=0.046), reinforcing the link between disseminated tumor burden and skeletal complications. Notably, treatment with ^131^I-Meta-iodobenzylguanidine (^131^I-MIBG) radionuclide therapy was associated with a reduced risk of SREs in both studies (significantly so in Laganà et al.), suggesting a potential protective effect. While Yokomoto et al. emphasized the importance of local skeletal invasion, Laganà et al. highlighted the prognostic impact of systemic dissemination. Together, these findings underscore the multifactorial nature of SRE risk in mPPGL, involving both local anatomical features and overall disease burden, and support a tailored approach to surveillance and bone-targeted therapy.

## 7 Quality of life

QoL in patients with mPPGL has not been systematically evaluated using standardized instruments. Existing studies have assessed QoL in PGL patients more broadly, without focusing specifically on those with BM. In a cohort of 174 patients with PGLs, van Hulsteijn et al. employed three validated questionnaires (HADS, MFI-20, and SF-36) and found that individuals with mPPGL reported significantly greater mental fatigue and lower general health perception compared to those with benign disease (61). Furthermore, patients with PGLs (regardless of metastatic status) report worse QoL compared to the general population, with increased fatigue, poorer physical functioning, and psychological and social impairments. These effects are more pronounced in individuals experiencing disease-related symptoms (61).

Although formal QoL assessments are lacking in studies focused exclusively on bone-metastatic PPGL, the available evidence strongly suggests a considerable impact. Retrospective studies have consistently highlighted the physical and functional burden associated with SREs, including complications such as bone pain, pathological fractures, and spinal cord compression (7, 36, 37). Metastatic bone lesions are particularly detrimental to QoL, as they can severely impair patients’ ability to carry out daily activities due to the significant pain and risk of fractures they cause (62). These events frequently result in reduced mobility, loss of independence, hospitalizations, and the need for intensive interventions, factors that are widely recognized as detrimental to QoL.

Further supporting this, von Moos et al., in a comprehensive review of metastatic bone pain across various advanced cancers (not limited to PPGL) (63), emphasized that skeletal complications significantly compromise QoL by contributing to persistent pain, impaired physical functioning, sleep disturbance, and emotional distress. The authors advocate for early and individualized management—including analgesia, radiotherapy, and bone-targeted agents—to mitigate the cumulative burden of BM on patient well-being (63). In addition, recent reviews in neuroendocrine tumors have highlighted that validated QoL instruments, such as the EORTC QLQ-C30, QLQ-GI.NET21, and Norfolk QoL-NET, include items specifically addressing bone or musculoskeletal pain, which represent a key determinant of reduced QoL in this population (64). While these data are derived from broader NET cohorts, they reinforce the relevance of systematically assessing bone pain and related symptoms in patients with mPPGL and BM.

Multidisciplinary management and the use of targeted therapies, such as bone resorption inhibitors and therapeutic radionuclides, can enhance symptom control and, in some cases, improve QoL in patients with BM. However, current evidence suggests that symptomatic relief does not always translate into prolonged survival, highlighting the need for individualized therapeutic goals based on clinical context and patient preferences (65). In this context, supportive care focused on pain control and the prevention of SREs is essential to preserving QoL in these patients (66).

In conclusion, the available evidence highlights the significant impact of BM on QoL in patients with PPGL. These findings underscore the importance of early supportive care, multidisciplinary management, and timely use of systemic and bone-targeted therapies to mitigate morbidity in this population.

## 8 Prognosis

The prognosis of patients with PPGL and BM is influenced by a range of clinical, histopathological, biochemical, and molecular features. However, most available data derive from studies on mPPGL in general, with limited evidence specifically addressing bone involvement. This section summarizes current knowledge regarding prognostic factors, survival differences related SREs, and the impact of selected treatments.

### 8.1 Prognostic factors associated with BM

In patients with mPPGL, several prognostic factors have been associated with worse outcomes. Independent predictors of shorter disease-specific survival (DSS) include age >30 years (Hazard Ratio [HR] 6.2; p < 0.0001), synchronous metastases (HR 4.9; p < 0.0001), elevated plasma methoxytyramine (MTY) levels (HR 2.4; p = 0.0010), and extensive metastatic burden (>5 lesions or >2 organs; HR 2.0; p = 0.0290) (67). Additional factors linked to poorer OS or more aggressive disease include older age at tumor diagnosis, male sex, larger primary tumor size, synchronous metastases, dopamine-secreting tumors, and absence of primary tumor resection (55). Histopathological features associated with distant metastases (including BM) are abdominal location (66.7%), tumor size >5.1 cm, >3 mitoses/10 HPF, SDHB loss, vascular and capsular invasion, nuclear pleomorphism, and confluent necrosis (68). Furthermore, older age at diagnosis and PASS score ≥7 have been associated with worse OS (24), while hormonal hypersecretion was the only independent predictor of poor prognosis in another cohort (HR 3.02; p=0.0004) (69).

While most prognostic data in mPPGL derive from general metastatic cohorts, a few studies have specifically addressed prognostic factors related to BM. One study reported that patients with PGL had a significantly higher risk of developing BM compared to those with PHEO (80% vs. 40%; p = 0.02) (37). The same study identified extraskeletal extension of spinal metastases—defined as invasion beyond the cortical bone—as a strong predictor of SRE development, occurring in 73% of patients with SREs versus none in those without (p = 0.001) (37). A large multicenter study focusing exclusively on patients with PPGL-related BM identified several prognostic factors (36). Lower risk of SREs was associated with ^131^I-MIBG therapy (HR 0.536; p = 0.027), absence of hepatic metastases (HR 0.638; p = 0.046), and, in univariate analysis, the presence of sclerotic bone lesions (HR 0.248; p = 0.007). Regarding OS, favorable outcomes were independently associated with age ≤48 years at PPGL diagnosis (HR 0.558; p = 0.002) and absence of liver metastases (HR 0.618; p = 0.034), while primary PHEO (as opposed to PGL) was linked to worse OS (HR 3.191; p = 0.001).

### 8.2 Survival differences in patients with or without SREs

In the multicenter study by Laganà et al. (36), SREs occurred in 31% of patients with bone-metastatic PPGL, most within the first four months after BM diagnosis. The presence of SREs was not significantly associated with worse overall survival (HR 0.808; p = 0.226), although these events represented an early and disabling complication in the clinical course of the disease. Similarly, in the cohort reported by Ayala-Ramírez et al. (7), overall survival was significantly shorter in patients with both bone and visceral metastases (median: 5 years) compared with those with only BM (12 years) or only non-osseous metastases (7.5 years; p = 0.005). However, survival differences specifically based on the presence or absence of SREs were not evaluated in that study. Collectively, these data suggest that while SREs do not independently impact overall survival, their early onset and disabling nature underscore the importance of prompt prevention and supportive management to mitigate morbidity and preserve QoL.

### 8.3 Impact of certain treatments on survival

In the study by Laganà et al. (36), certain treatments were significantly associated with improved OS in patients with mPPGL and BM. Specifically, treatment with bisphosphonates or denosumab (HR 0.598; p = 0.010) and ^131^I-MIBG therapy (HR 0.444; p = 0.001) were independently linked to longer OS. In contrast, chemotherapy was associated with worse prognosis, likely reflecting its use in patients with more aggressive disease. Similarly, in the cohort reported by Ayala-Ramírez et al. (7), systemic therapies and bone-targeted agents were associated with a significantly lower incidence of SREs: 79% of patients who did not develop SREs had received such treatments, compared to only 21% of those who experienced SREs (p < 0.0001). Although survival outcomes were not directly analyzed in that study, these findings support a potential protective role of antiresorptive and systemic therapies in mitigating skeletal complications and preserving QoL.

## 9 Imaging diagnosis

The evaluation of BM in PPGL requires a multimodal strategy that integrates anatomical and functional imaging, both for diagnosis and for assessing treatment response, which is commonly evaluated using RECIST criteria (4, 32, 70). However, no validated criteria currently exist for assessing skeletal response specifically in PPGL. The following section focuses on the role of imaging in the diagnosis and evaluation of BM in patients with mPPGL. In this section, we use the following abbreviations: computed tomography (CT), magnetic resonance imaging (MRI), and positron emission tomography/computed tomography (PET/CT).

### 9.1 Computed tomography

CT is valuable in the initial evaluation and follow-up of mPPGL patients with BM, providing detailed anatomic information on lesion site, size, extent, and complications (e.g., fractures or adjacent invasion) (71); the 2025 National Comprehensive Cancer Network (NCCN) guidelines recommend multiphasic CT (or MRI) with chest CT in suspected metastatic disease, correlated with functional imaging such as Somatostatin receptor (SSTR)-PET/CT or FDG-PET/CT (72). However, its sensitivity for BM detection is markedly lower than that of ^68^Ga-DOTATATE PET/CT: in a prospective cohort of 43 patients with spinal metastases, per-lesion detection reached 98.7% for 68Ga-DOTATATE PET/CT versus 44.8% for CT (p < 0.001) (73). These results should be interpreted with caution, as the cohort was relatively small and limited to spinal lesions, which may introduce spectrum bias. CT remains recommended in diagnostic protocols and follow-up, particularly to monitor the progression of known lesions and to assess structural response to therapy, although it may miss small or low-contrast lesions, especially in early stages or in bones with degenerative changes (73–75). Moreover, CT provides essential anatomical information for planning local interventions, including radiotherapy or surgery (31). For optimal staging, it should be complemented by functional imaging (73).

### 9.2 Magnetic resonance imaging

MRI is highly sensitive for detecting skeletal involvement in PPGL, particularly bone marrow lesions, allowing earlier detection than CT (76). Spinal MRI has shown a higher per-lesion detection rate than whole-body CT (80.6% vs. 44.8%), though it is outperformed by ^68^Ga-DOTATATE PET/CT (73). Beyond detection, MRI provides detailed characterization of marrow infiltration and cortical involvement, and is especially recommended for surveillance of patients with hereditary risk, such as carriers of SDHB, SDHD, or VHL mutations (1). Whole-body MRI (WB-MRI) demonstrates sensitivity and specificity (82% and 97%) comparable to MIBG scintigraphy (70). During follow-up, MRI is useful for monitoring treatment response through marrow signal changes indicating necrosis, fibrosis, or progression, and its lack of ionizing radiation makes it suitable for long-term surveillance, particularly in younger or genetically predisposed patients (76).

### 9.3 Scintigraphy with Iodine-^123^meta-iodobenzylguanidine or ^131^meta-iodobenzylguanidine

^123^I/^131^I-MIBG scintigraphy provides moderate sensitivity and high specificity for detecting metastatic PPGL, including bone involvement. In a multicenter study of 140 patients, ^123^I-MIBG achieved 82% sensitivity and 82% specificity, with higher performance in adrenal pheochromocytoma (88%) than in extra-adrenal paraganglioma (67%) (77). A recent cohort evaluating candidates for ^131^I-MIBG therapy reported 83–95% sensitivity and specificity, confirming its reliability when adequate norepinephrine-transporter uptake is present (70). Sensitivity declines in extra-adrenal, SDHB-mutated, or dedifferentiated tumors (78).

According to NANETS 2021 guidelines (6), SSTR-PET/CT is the first-line functional modality for staging, while MIBG is reserved for therapeutic selection and monitoring. ^131^I-MIBG can occasionally detect early bone lesions missed on conventional bone scans (79), but its incremental diagnostic value over CT/MRI is limited, and ^68^Ga-DOTATATE PET/CT generally provides superior sensitivity, particularly for skeletal disease (80).

Clinically, MIBG imaging remains essential for identifying patients eligible for ^131^I-MIBG therapy—positive uptake is a prerequisite—and for post-therapy follow-up, where imaging at 3–6 months predicts treatment response and survival (65). Thus, MIBG retains a focused yet important role in the modern management of PPGL, complementing PET-based functional imaging rather than replacing it.

### 9.4 PET/CT with ^68^Ga-labeled somatostatin analogues

^68^Ga-DOTATATE PET/CT is the most sensitive functional modality for detecting PPGL BM, owing to the high expression of somatostatin receptor subtype 2 (SSTR2) (73). In prospective comparative studies, it demonstrates a per-lesion detection rate of approximately 98–99%, markedly higher than ^18F-FDG PET/CT (49–85%, depending on genetic background). In patients with spinal BM, sensitivity reaches 98.7% per lesion and 100% per patient versus 72.0% and 90.2%, respectively, for ^18F-FDG PET/CT (73, 81, 82). This superiority is observed in both sporadic and SDHB-mutated metastatic PPGL, and is consistent with international guidelines recognizing ^68Ga-DOTATATE PET/CT as the preferred modality for detecting skeletal lesions (32). Compared with MRI and CT, it achieves higher detection rates (98.7% vs. 80.6% and 44.8%, respectively) and improves staging, therapy planning, and selection of candidates for Peptide Receptor Radionuclide Therapy (PRRT) (73, 80–84).

### 9.5 Positron emission tomography/computed tomography with fluorine-18-labeled fluorodeoxyglucose

^18^F-FDG PET/CT plays an important role in detecting and monitoring BM in PPGL, especially in metastatic disease and tumors with SDHB mutations. Its sensitivity exceeds 90% and surpasses CT and MRI for osseous lesions (32, 73, 74, 85). FDG uptake correlates with tumor aggressiveness, reaching lesion-based sensitivities of ~83% in SDHB-positive tumors, while being lower in SDHB-negative cases (32). This modality often reveals additional metastatic sites, influencing staging and treatment decisions (75, 86), and is useful in follow-up to assess response and progression (87). Despite its value, ^68^Ga-DOTATATE PET/CT generally offers superior sensitivity and is preferred when available (73, 75, 88).

### 9.6 Positron emission tomography/computed tomography with fluorine-18-labeled fluorodopa (^18^F-L-3,4-Dihydroxyphenylalanine)

The performance of ^18^F-FDOPA PET/CT depends on tumor subtype and genetic background. It shows high sensitivity for pheochromocytomas, both primary and metastatic, particularly in tumors without SDHB/SDHx mutations (83, 85, 89). For BM, however, its sensitivity is lower than that of ^68^Ga-DOTANOC PET/CT; for example, lesion-based detection reached 66.1% with ^18^F-FDOPA versus 96.2% with ^68^Ga-DOTANOC (89). Thus, in sympathetic PGL, mPPGL, or SDHx-related disease, ^68^Ga-labeled analogues are preferred (83, 85, 89). Nonetheless, ^18^F-FDOPA retains value in non-metastatic PHEOs and tumors without SDHx mutations, where its sensitivity may equal or exceed that of other functional modalities, and has shown greater accuracy than ^123^I-MIBG in recurrence or progression assessment (90).

### 9.7 ^18^F-Dopamine positron emission tomography/computed tomography

^18^F-FDA functions as a dopamine analogue and demonstrates enhanced affinity for the norepinephrine transporter system located in cellular membranes (91, 92). In a comparative study, ^18^F-FDA was able to detect a greater proportion of BM in affected patients, with a detection rate of 90%, outperforming bone scintigraphy (82%), CT/MRI (78%), and ^18^F-FDG PET/CT (76%) (93).

### 9.8 Other imaging techniques

Exploratory studies with ^18^F-fluorothymidine (^18^F-FLT) PET have shown limited proliferative activity in PPGL BM, with peri-tumoral uptake possibly reflecting reactive marrow changes (94). In clinical practice, patients with high-risk features—such as large primary tumors, extra-adrenal location, or SDHB mutations—require close imaging surveillance, with ^18^F-FDG PET/CT particularly useful in this subgroup (7). Overall, ^68^Ga-DOTATATE PET/CT remains the modality of choice for evaluating BM, complemented by MRI for anatomical assessment and follow-up, while CT and MIBG scintigraphy serve secondary or context-dependent roles (70, 73, 75, 83, 85, 95).

These imaging modalities not only facilitate early and accurate diagnosis but also have direct implications for patient management, therapeutic planning, and prognostication in mPPGL with bone involvement.

## 10 Management

Currently, there are no randomized controlled trials or open-label studies to establish evidence-based guidelines for the management of metastatic bone disease in mPPGL. Treatment strategies should be individualized for each patient, and whenever possible, decisions should be guided by the recommendations of a multidisciplinary endocrine tumor board (6).

**Figure 3:**
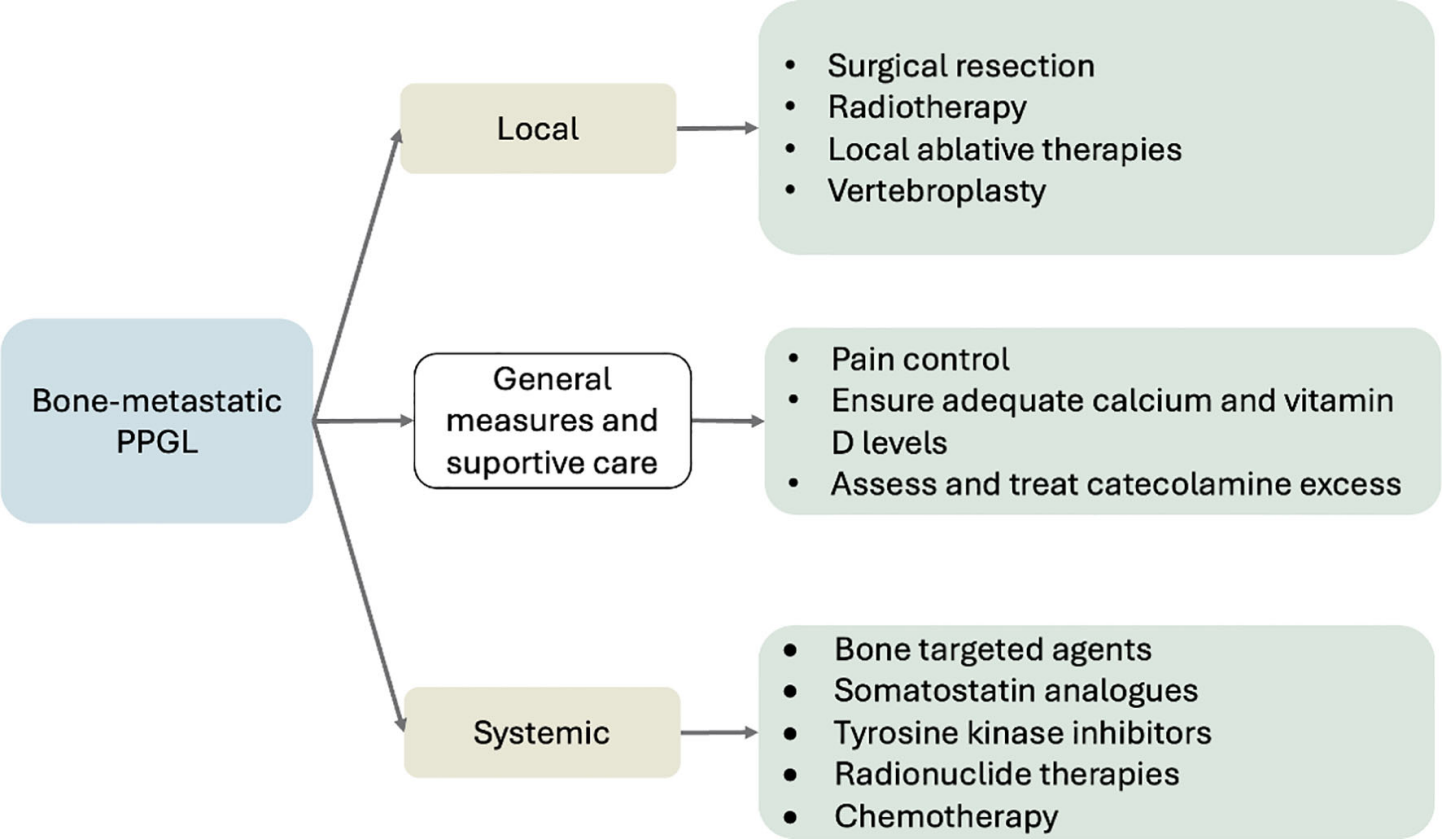
Overview of treatment strategies for BM in PPGL. Management integrates local (surgery, radiotherapy, ablation), systemic (radionuclide therapy, TKIs, chemotherapy, bone-targeted agents), and supportive measures (pain control, calcium/vitamin D optimization, catecholamine management). Adapted from NANETS (6), ESMO–EURACAN (54) and NCCN v2.2025 (72).

Therapeutic approaches can be broadly categorized into three main groups: (1) general measures and supportive care, (2) management of localized bone disease, and (3) systemic treatment strategies. An overview of these treatment domains is illustrated in **Figure 3**.

### 10.1 General measures and supportive care

When considering therapeutic strategies and/or goals of care for patients with BM in mPPGL—as with other malignancies—several key parameters should be evaluated: the extent of skeletal involvement (including bone structural integrity and the presence or absence of pathological fractures), the rate of progression of bone disease, the status of extra-skeletal metastatic sites, the presence or absence of symptoms (particularly pain), and the need to restore or preserve functional capacity and prevent disease-related complications (6).

General recommendations involve screening patients with PPGLs for secondary osteoporosis and providing sufficient calcium and vitamin D supplementation to maintain bone health and minimize fracture risk. Evaluating and addressing bone loss in individuals with secondary osteoporosis from PPGL should be a priority to improve outcomes and prevent fractures (96).

Prompt initiation of measures to control catecholamine excess is essential to prevent clinical deterioration and maintain QoL (97).

Bone pain management, prevention of SREs (e.g., with bisphosphonates or denosumab), and control of hypertension secondary to catecholamine excess are essential components of care in all patients (35, 98, 99).

### 10.2 Local disease

#### 10.2.1 Surgery

Surgical intervention plays a key role in the multidisciplinary management BM from PPGL. Indications for surgery may be guided by clinical decision-making tools and individualized assessment of factors such as the presence or risk of pathological fractures, intractable pain, or impending neurological compromise due to spinal cord or nerve root compression (6). In cases of spinal cord compression, prompt surgical decompression is indicated, typically followed by radiotherapy to achieve durable local control and preserve neurological function (97).

Beyond the management of skeletal complications, surgical resection of the primary catecholamine-producing tumor also has systemic benefits. Bone health is often compromised in patients with PPGL due to the effects of catecholamine excess on bone metabolism. Therefore, it is important to evaluate both BMD and trabecular bone score (TBS), as both parameters have been shown to improve following tumor resection (2, 52). A recent meta-analysis demonstrated that patients with PPGL exhibit lower TBS values and elevated levels of bone turnover markers such as cross-linked C-telopeptide of type I collagen (CTx) and bone-specific alkaline phosphatase (BS-ALP). Surgical removal of the tumor via adrenalectomy led to measurable improvements in these parameters, underscoring the metabolic benefits of tumor control (45, 96, 100).

In addition to conventional resection and stabilization procedures, innovative surgical techniques have also been reported. Kitagawa et al. described a case of PGL-related BM successfully treated with liquid nitrogen-frozen autologous bone reconstruction. Bone fusion was achieved without local recurrence, and the patient’s QoL remained preserved. This approach highlights promising advances in the surgical management of metastatic bone lesions (101).

#### 10.2.2 Radiation therapy

RT plays a crucial role in managing PPGL-related BM, particularly when systemic treatments are ineffective or unavailable, and patients present with symptoms. It offers rapid pain relief and contributes to localized tumor control, either as a standalone option or following surgical intervention (6, 97). Postoperative radiotherapy is a viable option in patients with spinal cord compression due to osseous metastases, helping prevent recurrence and preserve neurological function (97).

Overall, RT administered at doses exceeding 40 gray has been shown to achieve local tumor control and symptomatic relief in metastatic lesions across various sites, including soft tissue, liver, and bone (102, 103). However, some authors have also reported successful outcomes with lower radiation doses, such as 20 Gy (58).

External beam radiation therapy (EBRT) has been employed for both symptomatic and local control of these lesions. In the study by Vogel et al. (104), radiographic outcomes were assessed, showing that 86.7% of lesions treated with EBRT achieved stable disease, while 13% demonstrated progression. Additionally, symptomatic relief was reported in 81.1% of treated lesions. Although specific outcomes for bone lesions in the context of combination therapy with ^131^I-MIBG were not detailed, these findings suggest that EBRT may be effective for achieving local control of osseous metastases in these malignant tumors.

According to the NANETS consensus recommendations, EBRT is recognized as a valuable palliative modality for symptomatic BM, particularly in the presence of pain or risk of pathological fracture. While data on tumor control remain limited, its therapeutic benefit centers on symptom alleviation and structural stabilization (6). This recommendation is also echoed in the updated National Comprehensive Cancer Network (NCCN 2025) guidelines, which endorse EBRT as part of the multimodal management of symptomatic PPGL-related BM when surgery is not feasible (72). Importantly, the NANETS and NCCN 2021 guidelines recommend pre-radiotherapy alpha-adrenergic blockade in hormonally active PPGLs to prevent catecholamine-related complications, with expert consensus supporting its use in biochemically active cases (31, 72).

Furthermore, advanced techniques such as hypofractionated intensity-modulated radiotherapy (IMRT) have also shown promising results. In particular, IMRT has demonstrated efficacy in achieving local control of advanced or recurrent PPGL lesions, with significant improvement in catecholamine-related symptoms reported in 91% of cases. Although not specific to BM, these findings support the broader therapeutic role of modern RT modalities in this disease (105).

Patients should be closely monitored during RT, as RT-induced inflammation of metastatic lesions may, in rare cases, trigger massive catecholamine release and hypertensive crisis (106).

#### 10.2.3 Local ablative therapy

A variety of nonsurgical, image-guided ablative techniques are accessible for the treatment of metastatic lesions, including radiofrequency ablation (RFA), cryoablation, and percutaneous ethanol injection (97). These therapies have demonstrated efficacy in limited studies and may serve as treatment options for patients who are unsuitable for surgery or as non-surgical alternatives (6).

Percutaneous tumor ablation is most effective in patients with one or a few relatively small lesions (ideally <3–4 cm). With appropriate periprocedural management, ablation can be safely performed at various metastatic sites, including soft tissue, bone, lung, and liver (107–112).

In a retrospective study involving 31 patients with metastatic PPGL, 123 lesions were treated with percutaneous ablation—most commonly using RFA, cryoablation, or ethanol injection. At a median follow-up of 60 months, radiographic local control was achieved in 86% of evaluable lesions, and 92% of procedures resulted in symptomatic improvement. Two-thirds of the interventions were complication-free, and most adverse events were mild to moderate (111).

Another study demonstrated that interventional radiology procedures can delay the onset of severe SREs in patients with mPPGL, particularly in those with moderate bone tumor burden—defined as five or fewer lesions or none exceeding 2 cm—highlighting their value in multidisciplinary management (113).

Both the 2021 NANETS and the updated 2025 NCCN guidelines support the use of local ablative therapies in carefully selected patients with mPPGL, particularly when surgical resection is not feasible (6, 72). NANETS emphasizes their utility for symptom control, reduction of tumor burden, and prevention of local complications in symptomatic or progressive disease (6). In alignment with these recommendations, the 2020 ESMO–EURACAN guidelines also acknowledge the role of locoregional interventions (such as RFA, cryoablation, microwave ablation, and chemoembolization) in selected patients with metastatic PPGL, especially for symptom relief and prevention of bone-related complications, including SREs (54). Collectively, these recommendations underscore the relevance of ablative therapies as part of a multidisciplinary strategy focused on localized disease control and the preservation of patient QoL.

#### 10.2.4 Vertebroplasty

Vertebroplasty, although supported by limited clinical evidence, has demonstrated therapeutic benefit and may serve as a feasible alternative for patients with metastatic PPGL who are not suitable candidates for conventional surgical intervention (6). Among localized interventions aimed at reducing the incidence of SREs, vertebroplasty represents a targeted option for stabilizing structurally compromised weight-bearing bones affected by lytic metastatic lesions, contributing to symptomatic relief and mechanical reinforcement (113).

### 10.3 Systemic disease

In patients with mPPGL presenting with diffuse or widespread disease, particularly when bone is not the only organ involved, as is most often the case (7), surgical or locoregional therapies are generally not feasible. In such scenarios, management strategies typically rely on either systemic therapies or active surveillance, depending on the clinical context and disease burden. Most available studies have not specifically evaluated or reported the efficacy of systemic treatments based on the site of metastatic involvement (e.g., bone). This remains a challenge to be addressed in future research and highlights the current need to individualize treatment strategies according to patient-specific clinical contexts.

For patients with asymptomatic, indolent disease, observation is preferred over systemic therapy, as potential side effects may outweigh the benefits. Treatment should be initiated if symptoms develop or the disease progresses (114).

Systemic therapies are particularly appropriate for extensively metastatic, progressing, or symptomatic disease when localized approaches cannot adequately address the overall tumor burden (6). In this context, various of the systemic options might delay or prevent the onset of SREs (7). CVD chemotherapy is recommended particularly in rapidly progressing cases or those with a high visceral tumor burden requiring tumor shrinkage, due to its high ORRs (4, 6, 115, 116). For patients with moderate to rapidly progressive disease ineligible for chemotherapy, treatment relies on molecular targeted therapy, focusing on key molecules in cancer growth and survival. The choice depends on radionuclide imaging positivity: SSTR2-positive cases are treated with PRRT, while MIBG-positive cases receive ¹³¹I-MIBG therapy, using either high- or low-specific activity formulations (4, 115–119). In cases with no positivity in radionuclide imaging, the treatment of choice is Tyrosine kinase inhibitors (TKIs) such as Sunitinib or Cabozantinib. Finally, patients with Slowly progressive disease, and SSTR positive disease, Somatostatin analogs (SSAs) could be an option (6, 97).

**Figure 4:**
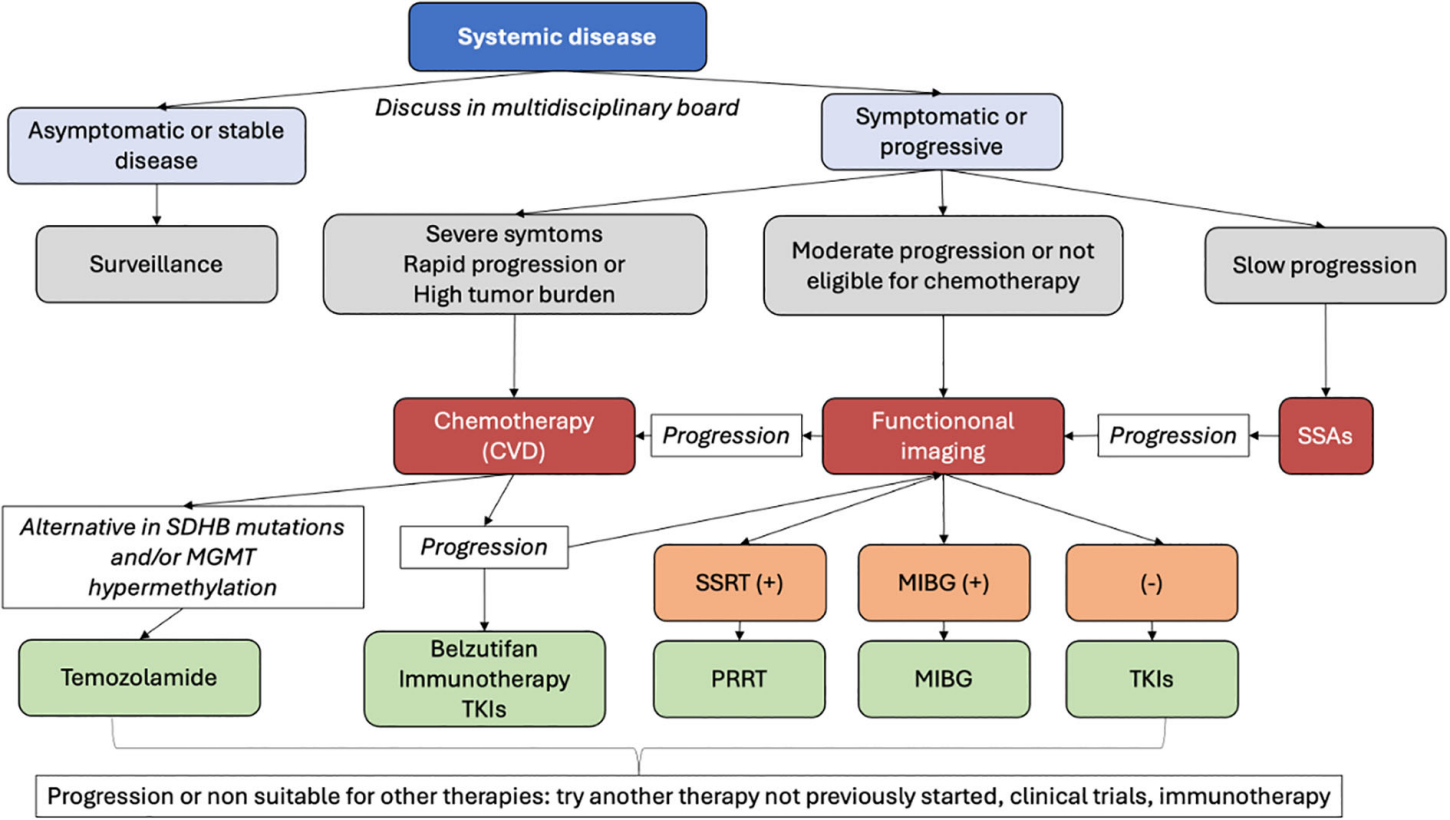
Management options for systemic disease in mPPGL.

A proposed algorithm for management options in systemic disease can be found at **Figure 4**. A summary of systemic treatment regimens, including dosages and key adverse effects, is provided in **Table 2**.

**Table 2:** Systemic therapies for mPPGL: commonly dosing regimens used and main adverse events reported.

Treatment	Typical dosage/regimen	Main adverse effects
Chemotherapy (CVD: cyclophosphamide, vincristine, dacarbazine) (35, 99, 169–171)	Cyclophosphamide 750 mg/m² IV (day 1); Vincristine 1.4 mg/m² IV (max 2 mg, day 1); Dacarbazine 600 mg/m² IV (days 1–2). Cycle every 21–28 days	Myelosuppression, nausea/vomiting, alopecia, peripheral neuropathy (vincristine), fatigue, rare hemorrhagic cystitis or secondary malignancies
Temozolomide monotherapy (134, 172)	– 150–200 mg/m² orally once daily on days 1–5 of a 28-day cycle; dose escalation to 200 mg/m² if tolerated without significant myelosuppression	hematologic (lymphopenia, thrombocytopenia, neutropenia, anemia; grade 3–4 cytopenias in ~14–17%), fatigue, nausea, vomiting, constipation, alopecia, headache, anorexia. Less common but serious toxicities include hepatotoxicity, opportunistic infections (notably Pneumocystis jirovecii pneumonia in prolonged lymphopenia, requiring prophylaxis), and rare secondary malignancies (MDS/AML).
Belzutifan (139, 140)	120 mg orally once daily (adults and ≥12 years, ≥40 kg); 80 mg daily if <40 kg. Continue until progression or unacceptable toxicity; dose adjustments as needed	Anemia (very common, grade 3 in ~22 %), fatigue, musculoskeletal pain, dyspnea, hypoxia, and hypertension (each clinically significant). Other frequent events include nausea, constipation, dizziness, headache, edema, lymphopenia, and elevated liver enzymes. Serious adverse events occurred in ~11 % of patients in the phase 2 trial and ~36 % in pooled safety data from the prescribing information; fatal events were rare (< 1 %).
Sunitinib (141, 143)	37.5 mg orally once daily, continuous dosing until progression or unacceptable toxicity (alternative: 50 mg daily, 4 weeks on/2 weeks off).	fatigue/asthenia, hypertension (overall up to 29%, grade 3–4 in 7–13%), thrombocytopenia, hepatotoxicity (rarely fatal), and cardiovascular events (CHF, QT prolongation). Other risks: bleeding, proteinuria, thyroid dysfunction, hypoglycemia, osteonecrosis of the jaw, impaired wound healing, and GI symptoms. Rare but serious: RPLS, thrombotic microangiopathy, tumor lysis syndrome.
Cabozantinib (145, 173)	60 mg orally once daily on an empty stomach until progression or unacceptable toxicity (40 mg daily if <40 kg).	Diarrhea, fatigue, palmar-plantar erythrodysesthesia (PPE), decreased appetite, hypertension, nausea, vomiting, weight loss, constipation, and mucositis. Grade 3–4 toxicities include hypertension (~21%), fatigue (~13%), diarrhea (~11%), PPE, mucositis, nausea, anorexia, neutropenia, and lymphopenia. Serious risks: hemorrhage, GI perforation/fistula, thrombotic events, hepatotoxicity, adrenal insufficiency, proteinuria, osteonecrosis of the jaw, impaired wound healing, RPLS, thyroid dysfunction, and hypocalcemia
PRRT (^177^Lu-DOTATATE) (146–152)	7.4 GBq (200 mCi) IV every 8 weeks × 4 cycles (cumulative 29.6 GBq), with concurrent long-acting octreotide 30 mg IM after each cycle and monthly thereafter.	Myelosuppression (lymphopenia most common; grade ≥3 up to 45%), thrombocytopenia, neutropenia, anemia; renal toxicity (rare with amino acid protection); transient hepatotoxicity; secondary MDS/AML (rare, late onset); catecholamine release syndrome (~17% in PPGL, requires antihypertensive precautions); infusion/hypersensitivity reactions; neuroendocrine crises (flushing, diarrhea, bronchospasm, hypotension); common mild AEs: nausea, vomiting, headache, fatigue. Dose reductions (to 3.7 GBq) or discontinuation may be needed for significant hematologic/renal toxicity.
131I-MIBG (154, 174, 175)	Standard dose 150–200 mCi (5.55–7.4 GBq) IV per session; repeatable at intervals of several months. High-dose regimens (up to ~12 mCi/kg or 800–1,160 mCi) may be used in select cases, sometimes requiring stem cell support	Hematologic toxicities (grade 3–4 neutropenia, thrombocytopenia, leukopenia, lymphopenia; risk increases with high or repeated dosing), rarely irreversible marrow aplasia. Non-hematologic effects include nausea, appetite loss, constipation, and acute hypertension during/after infusion. Less common serious events: ARDS, infection, MDS/AML, hypogonadism, renal toxicity, and thyroid dysfunction (preventable with blockade). Most adverse effects are manageable and reversible
Octreotide (162, 176)	Doses ranged from 20–30 mg monthly.	Hyperglycemia, gastrointestinal symptoms, and diarrhea leading to discontinuation
Lanreotide	limited data	limited data
Immunotherapy (165)	Pembrolizumab 200 mg IV every 3 weeks; Nivolumab 240 mg IV every 2 weeks or 3 mg/kg IV q2w; Ipilimumab 1 mg/kg IV every 6 weeks (in combination)	Fatigue, rash, diarrhea, hypothyroidism, decreased appetite. Immune-related toxicities include thyroid dysfunction, adrenal insufficiency, hepatitis, colitis, and pneumonitis.
BTA (Denosumab, Zoledronic acid and Pamidronate) (6, 7, 63, 124, 125)	Denosumab: 120 mg SC every 3 months (commonly used); in some reports, monthly dosing has also shown pain relief in some cases	Denosumab: Hypocalcemia, osteonecrosis of the jaw, atypical femoral fractures, and rebound vertebral fractures after discontinuation
	Zoledronic acid: 4 mg IV every 3–4 weeks	Biphosphonates: Hypocalcemia, osteonecrosis of the jaw, atypical femoral fractures, and rebound vertebral fractures upon abrupt discontinuation, nephrotoxicity, flu-like symptoms.
	Pamidronate: 90 mg IV every 3 months.	

*Doses are reported as used in metastatic PPGL in general; specific data for bone metastases are limited or unavailable in some cases.

#### 10.3.1 Bone targeted agents

Since the late 1990s, parenteral bisphosphonates have been employed to prevent SREs in patients with lytic BM from malignancies such as breast cancer and multiple myeloma (120, 121). Furthermore, analyses of randomized studies in patients with castration-resistant prostate cancer (122) and a meta-analysis in those with metastatic castration-sensitive prostate cancer (123) have shown that bone-targeted therapies are associated with improved prognosis.

Although the direct evidence supporting the use of antiresorptive agents for BM in mPPGL is limited, extrapolation from studies in other solid tumors provides a rationale for their use. The NANETs guidelines (6) recommend considering antiresorptive or BTA—such as denosumab, zoledronic acid, or pamidronate—in patients with focally extensive or disseminated disease, irrespective of systemic treatment. These agents have demonstrated efficacy in reducing the incidence of SREs, including pathologic fractures and the need for radiotherapy, in patients with metastatic disease from breast, prostate, and lung cancers. Among available options, denosumab has been suggested to offer the most significant clinical benefit (124). In patients with PPGL specifically, denosumab has been associated with notable improvements in severe bone pain (7).

Commonly used regimens (derived from clinical experience in other malignancies)include denosumab 120 mg administered subcutaneously every three months or zoledronic acid 4 mg intravenously every three months, with treatment generally continued throughout the course of disease progression (63). Importantly, in a case series involving patients with PPGL, two individuals with severe bone pain achieved complete symptom relief following monthly administration of denosumab at 120 mg subcutaneously (7). Additionally, an exceptional response to zoledronic acid has been reported in a patient with bone-predominant metastatic PGL who had experienced multiple relapses. After one year of therapy, complete resolution of lymphadenopathy and somatostatin receptor–avid lesions was observed by PET-CT with 68Ga-DOTATATE, and disease stability was maintained for several years thereafter (125). Supporting this, primary culture studies of PPGL cells have also demonstrated a direct antitumor effect of zoledronic acid, suggesting a possible therapeutic benefit beyond SRE prevention.

Although direct evidence for the resolution of hypercalcemia with bisphosphonates in PPGL-related BM is lacking, their benefit is well established in malignant hypercalcemia across cancers. Zoledronic acid (4 mg IV) is the preferred bisphosphonate due to greater efficacy and longer duration compared to pamidronate (90 mg IV), achieving calcium normalization in ~88% of cases and sustaining the response for longer periods (126, 127). Therefore, their use can be reasonably extrapolated to PPGL patients with BM who present with hypercalcemia.

If disease progression occurs while on one BTA, or new skeletal lesions develop, switching to an alternative antiresorptive therapy should be considered. These agents, however, are not without risks. Reported complications include hypocalcemia, osteonecrosis of the jaw, atypical femoral fractures, and rebound vertebral fractures upon abrupt discontinuation (128). To reduce the likelihood of adverse events, it is recommended to ensure adequate calcium and vitamin D levels and to perform a thorough dental evaluation prior to initiation of therapy (6).

Vitamin D deficiency has been linked to worse overall survival in gastroenteropancreatic NETs and a higher incidence of bone metastases in lung NETs. In addition, patients with intestinal NETs receiving SAs often develop vitamin D deficiency, highlighting the need for nutritional monitoring (64). Although data are lacking in PPGL, these findings suggest that vitamin D status may influence skeletal outcomes in NETs.

Prospective studies investigating the use of BTA in patients with PPGL at the time of BM diagnosis would provide much-needed evidence to guide clinical decision-making in this rare disease.

#### 10.3.2 Citotoxic chemotherapy - cyclophosphamide, vincristine, dacarbazine

Data on the efficacy of CVD chemotherapy specifically regarding bone-related outcomes in patients with mPPGL are limited. Clinically, in the study by Ayala-Ramirez et al. (2013) (7), although specific radiologic responses of BM to CVD were not reported, systemic treatment (including chemotherapy and antiresorptives) was associated with a reduced risk of SREs. From a biochemical and imaging standpoint, additional evidence has been reported in case studies and small series. In a case described by Patel et al. (1995) (129), a patient with a bulky retroperitoneal tumor and a solitary T12 BM achieved a partial response to CVD chemotherapy. After surgical resection of the primary tumor, biopsy of the vertebral lesion showed no residual disease, indicating resolution of the BM (129). Similarly, in the study by Huang et al. (2008) (130), 67% of patients had BM. Bone scan improvement was observed in four patients, all of whom also had radiographic and biochemical responses to CVD, suggesting a concordant therapeutic effect in bone lesions among responders. In another study, Tanabe et al. (2013) (131): decreased ¹²³I-MIBG uptake was observed in BM of two patients, despite no change in lesion size on CT. This suggests a potential functional response to CVD chemotherapy not reflected by anatomical imaging.

In mPPGL in general (not specifically limited to BM), international guidelines and systematic reviews position chemotherapy as a treatment option reserved for patients with rapidly progressive disease, high tumor burden, symptomatic, or when the use of targeted therapies such as ^131^I-MIBG or PRRT is not feasible, or in tumors with a high proliferative index (35, 72). NANETS guidelines highlight its benefit for tumor control, especially in SDHB-mutated cases (6).

Treatment with СVD is generally well tolerated. The most common toxicities are mild myelosuppression, periperal neuropathy, and gastrointestinal toxicity (6, 132).

#### 10.3.3 Chemotherapy - temozolomide

Temozolomide is indicated in mPPGL, particularly in patients with SDHB mutations or MGMT promoter hypermethylation. It is considered for progressive, symptomatic, or unresectable disease due to its favorable toxicity profile and potential for tumor and biochemical response, although most data refer to metastatic PPGL in general and not specifically to BM (6, 133, 134).

#### 10.3.4 Chemotherapy - other regimens

Although not specifically focused on BM, other regimens have demonstrated utility in metastatic PPGL, including gemcitabine (135), gemcitabine combined with paclitaxel (136), docetaxel (137) and paclitaxel monotherapy (138). Carboplatin and etoposide–based regimens have also been used as palliative therapy in cases of advanced PPGL (41). However, further evidence is needed to confirm their efficacy.

#### 10.3.5 Belzutifan

Belzutifan, a selective hypoxia-inducible factor-2α (HIF-2α) inhibitor, has been approved by the FDA for the treatment of advanced or metastatic pheochromocytoma and paraganglioma (mPPGL) in patients aged 12 years and older (139). This indication is supported by the phase 2 international LITESPARK-015 trial (140), which enrolled 72 patients with unresectable or metastatic disease and reported an objective response rate of 26% and a disease-control rate of 85%, with a median progression-free survival of 22.3 months. Most responses were durable, and treatment was generally well tolerated. However, specific outcomes in patients with bone metastases remain to be defined.

#### 10.3.6 Tyrosine kinase inhibitors

##### 10.3.6.1 Sunitinib

The strongest evidence for the use of sunitinib in mPPGL in general comes from the FIRSTMAPPP trial, which included 78 patients with progressive disease. Sunitinib significantly improved 12-month progression-free survival (PFS) (36% vs. 19%) with manageable toxicity. Although bone-specific outcomes were not detailed, many patients had skeletal disease, and the overall benefit is considered applicable (141). Additional prospective and retrospective studies, such as SUTNET and SNIPP, confirm sunitinib’s activity in advanced disease, showing disease control rates over 80%, objective responses around 15%, and PFS between 8.9 and 14 months, including clinical and radiologic benefit in BM, though without dedicated subgroup analyses (142, 143).

##### 10.3.6.2 Cabozantinib

Cabozantinib is an antiangiogenic multi-tyrosine kinase inhibitor that has demonstrated improvement in bone health in patients with various malignancies. A phase II trial (144)evaluated cabozantinib in patients with BM from non-breast, non-prostate solid tumors. While the study did not include patients with PPGL, it provides evidence of cabozantinib’s activity in BM. No SREs occurred, and significant reductions in bone turnover markers (serum C-telopeptide and N-telopeptide) were observed. The drug was generally well tolerated and showed antitumor activity.

In the setting of PPGL, Cabozantinib presents a viable substitute for chemotherapy in patients with mPPGLs exhibiting moderate to rapid disease progression. A phase II clinical study involving 17 individuals diagnosed with malignant PHEO or PGL showed that cabozantinib achieved an objective response rate (ORR) of 25% (145).

#### 10.3.7 Radionuclide therapies

##### 10.3.7.1 Peptide receptor radionuclide therapy

PRRT with Lutetium-177-DOTATATE (^177^Lu-DOTATATE) has demonstrated significant clinical efficacy in patients with metastatic NETs involving the skeleton. In contemporary cohorts, the objective response rate for BM ranges from 23% to 31%, with disease control rates exceeding 75%. Notably, substantial symptomatic benefit has been reported, including complete resolution of bone pain in approximately 39% of patients and partial improvement in more than 50% (146–148). The median PFS in patients with BM ranges from 27 to 33 months, while median OS ranges from 35 to 46 months, even among subgroups with extensive skeletal disease (146–148). The extent of skeletal metastatic burden does not appear to significantly impact the objective response rate (146). Regarding safety, SREs (such as fractures, spinal cord compression, etc.) occur in approximately 20% of patients with BM (146). QoL improves significantly following treatment, even in patients with extensive skeletal involvement (147).

Although BM in mPPGL have not been specifically studied, patients with moderately progressive mPPGL expressing SSTRs may benefit from PRRT with lutetium ^177^Lu- DOTATATE. This therapy has demonstrated clinical and biochemical efficacy in functional mPPGL, with a favorable safety profile. Treatment benefits include tumor shrinkage and symptom relief, with eligibility determined by SSTR expression on ^68^Ga-DOTATATE PET-CT (149–152).

##### 10.3.7.2 ^131^I-MIBG

Alongside Belzutifan, ^131^I-MIBG remains the only U.S. Food and Drug Administration (FDA)-approved systemic therapy for patients with mPPGL (4, 116). Clinical evidence supports the use of iobenguane ^131^I-MIBG in PPGL-related BM, provided there is demonstrable MIBG avidity on functional imaging. High-specific-activity (HSA) MIBG has shown disease control rates of 55–86%, with partial responses in 20–30% and disease stabilization in most patients, including those with skeletal involvement (65, 72, 153–158).

Importantly, Laganà et al. (36) study demonstrated that treatment with MIBG radionuclide therapy was associated with a reduced risk of SREs in both univariable and multivariable analyses, supporting its role as a protective therapeutic strategy in patients with PPGL.

**Figure 5:**
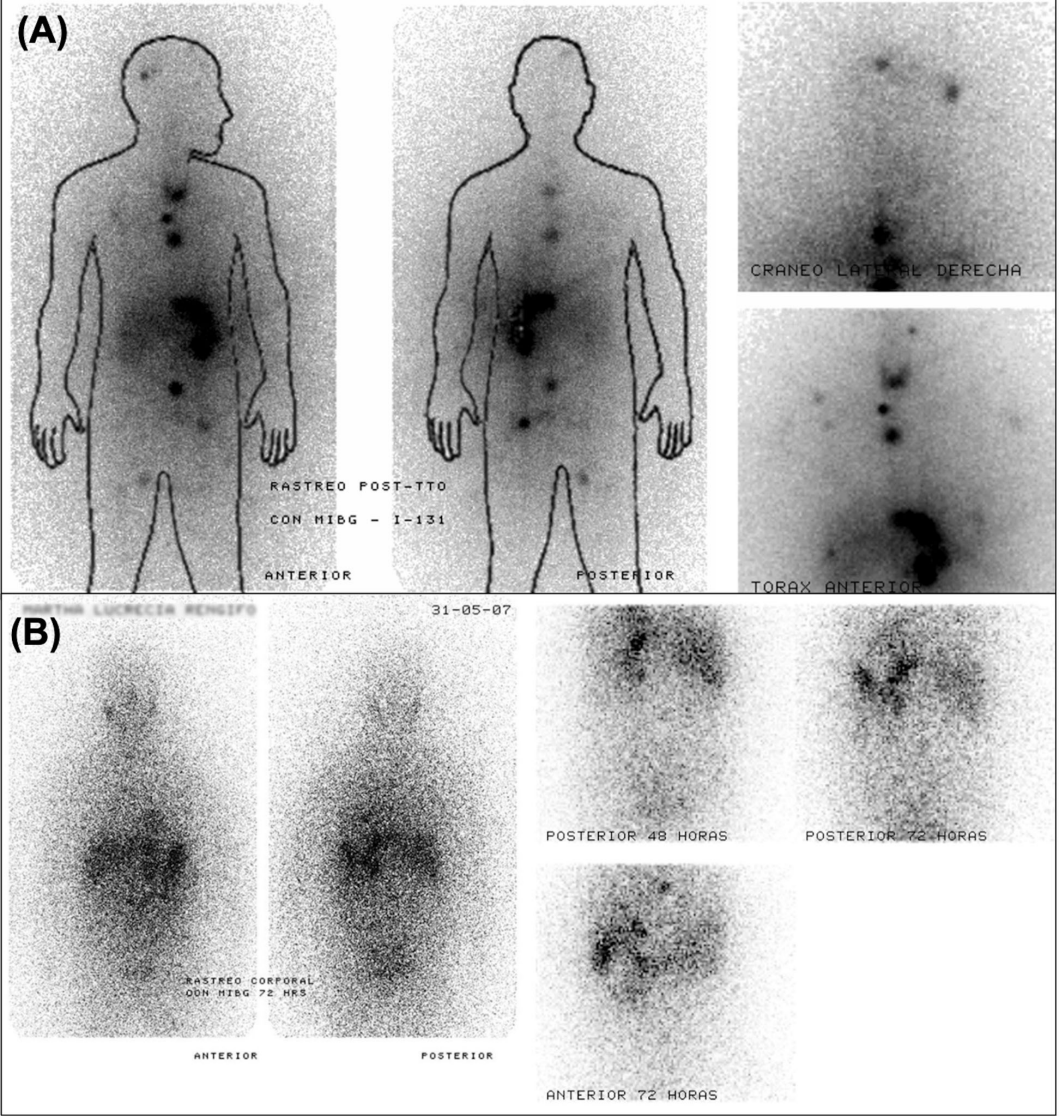
Serial ¹³¹I-MIBG scintigraphy demonstrating partial response to therapy in a patient with metastatic PPGL. **(A)** Post-therapeutic scan acquired eight days after administration of 300 mCi of ¹³¹I-MIBG shows radiotracer uptake in the skull, mediastinum, retroperitoneum, left internal iliac region, and right inguinal area. **(B)** Follow-up scan six months later, using 5 mCi of ¹³¹I-MIBG, reveals resolution of most lesions, with persistent uptake only in the mediastinum and liver.

**Figure 5** shows a representative case with partial resolution of previously identified metastatic lesions following high-dose ¹³¹I-MIBG, as demonstrated by comparative pre- and post-treatment scans. This observation is consistent with isolated case reports of vertebral metastases responding to repeated MIBG therapy, supporting the potential for disease control in bone lesions under specific circumstances (159).

Main toxicities include reversible myelosuppression (grade 3–4 cytopenias in up to 80%) (156), as well as nausea, fatigue, hypothyroidism, and gonadal failure at high cumulative doses (154, 155). Severe renal or cardiovascular toxicity is uncommon with HSA-MIBG (153, 155).

#### 10.3.8 Somatostatin analogues

In NETs, SSAs represent a cornerstone of therapy due to their efficacy in symptom control, tumor stabilization, and favorable safety profile, even at escalated doses (160). Evidence in mPPGL, however, remains limited. While early studies did not demonstrate significant benefit (161, 162), more recent reports suggest sustained disease control, with a 100% disease control rate at three months among six treated patients, median time to progression not reached, and 75% progression-free survival at 23 months (4). Lanreotide has similarly shown disease stabilization in small series (163) and is currently under evaluation in a dedicated clinical trial (NCT03946527). Potential benefit appears greater in tumors with high SSTR2 expression, such as SDHB-mutated PPGLs (163). Although data regarding bone outcomes are lacking, SSAs may be considered in selected patients with functional disease, slow progression, or low tumor burden.

#### 10.3.9 Immunotherapy

Evidence for immunotherapy in PPGL-related BM is limited, with no strong clinical trial data. Current insights mainly come from preclinical studies and case reports suggesting potential benefit of immune checkpoint inhibitors. Combined immunotherapy in mouse models has shown reduced metastases through activation of innate and adaptive immunity, with CD8+ cells playing a key role (164). However, these findings have not been confirmed in humans and cannot yet be applied to clinical practice. Immune checkpoint inhibitors are being studied in ongoing trials, with some case reports available, but clinical experience remains limited and current data are insufficient for clear recommendations (116, 165–167). Additionally, these tumors show low PD-L1, minimal immune cell infiltration, and poor antigenicity, indicating limited responsiveness to standard immunotherapy (168). Guidelines view immunotherapy as experimental in clinical trials, not standard care for BM in PPGL.

## 11 Conclusion

**Figure 6:**
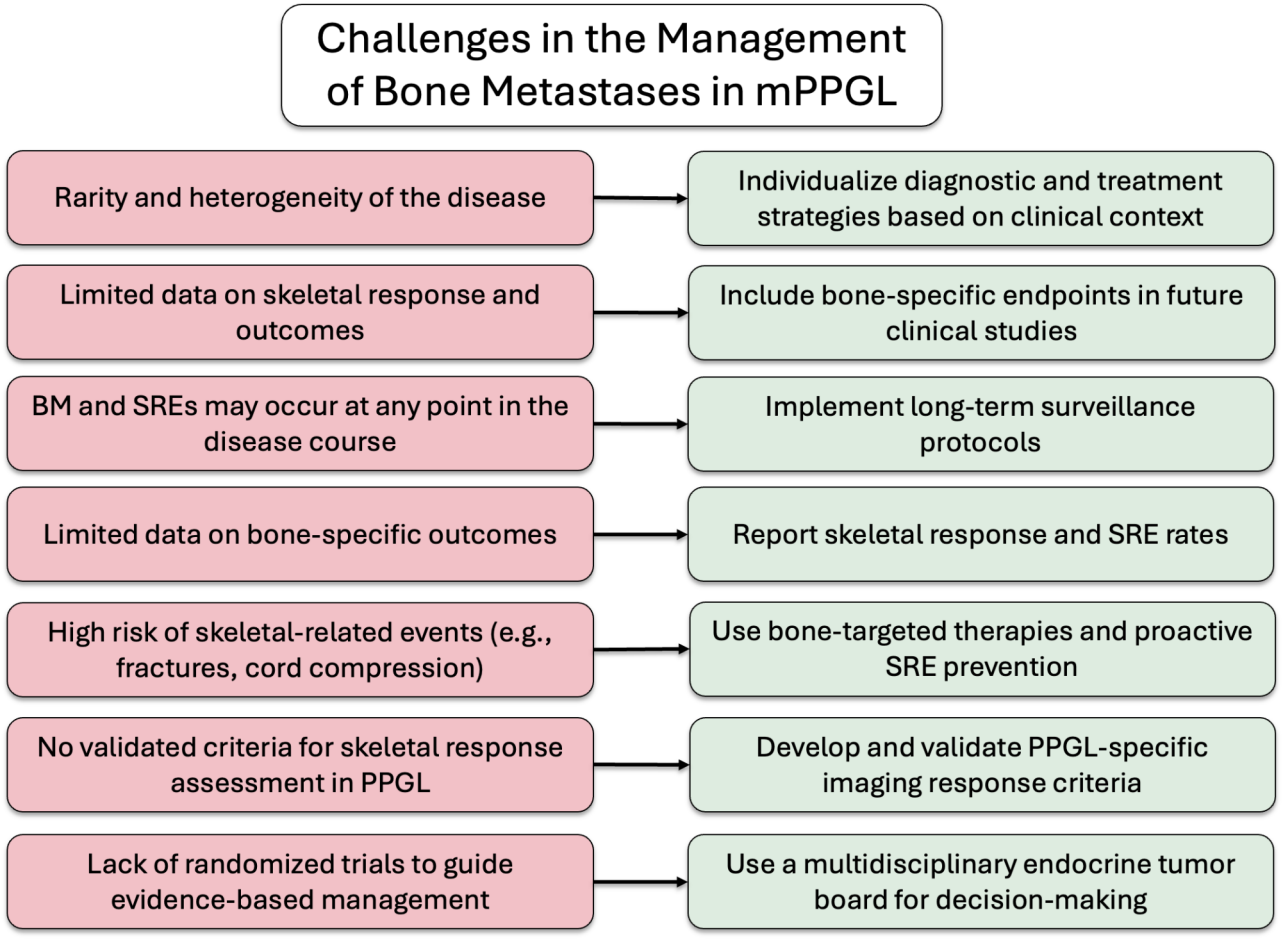
Key challenges and proposed solutions in the management of BM in mPPGL.

In summary, BM are common in patients with mPPGL and are associated with a high incidence of SREs, underscoring the importance of long-term monitoring and proactive management. Early identification and appropriate treatment of bone involvement are essential to improve both QoL and OS. The potential for late-onset metastatic spread, even years after the initial diagnosis, should always be considered. An integrated, multidisciplinary approach (encompassing endocrinology, oncology, nuclear medicine, palliative care, radiotherapy, orthopedic surgery, and neurosurgery) is fundamental to providing optimal care for these complex patients. To aid clinical decision-making, a proposed algorithm outlining challenges and possible solutions is presented in **Figure 6**.

## Glossary

18F-FDA: Fluorodopamine
BM: Bone Metastases
BMD: Bone Mineral Density
CT: Computed Tomography
EBRT: External Beam Radiotherapy
FDA: U.S. Food and Drug Administration
FDG: Fluorodeoxyglucose
FDOPA: Fluorodopa
FLT: 18F-Fluorothymidine
HSA: High-Specific-Activity
IMRT: Intensity-Modulated Radiotherapy
MIBG: Meta-Iodobenzylguanidine
mPPGLs: Metastatic Pheochromocytoma and Paraganglioma
MRI: Magnetic Resonance Imaging
NCCN: National Comprehensive Cancer Network
NET: Neuroendocrine Tumors
OS: Overall Survival
PET/CT: Positron Emission Tomography/Computed Tomography
PFS: Progression-Free Survival
PGLs: Paragangliomas
PHEOs: Pheochromocytomas
PPGL: Pheochromocytomas and Paragangliomas
PRRT: Peptide Receptor Radionuclide Therapy
QoL: Quality of Life
SDHB: Succinate Dehydrogenase Complex Subunit B
SDHD: Succinate Dehydrogenase Complex Subunit D
SSAs: Somatostatin analogs
SRE: Skeletal-Related Events
SSTR: Somatostatin Receptor
TKIs: Tyrosine Kinase Inhibitors
VHL: von Hippel–Lindau
