# Supplementary material for: Bone metastases in pheochromocytomas and paragangliomas: a narrative review of presentation, diagnosis and management
Source: Front Endocrinol (Lausanne). 2025 Nov 5;16:1671486. doi: 10.3389/fendo.2025.1671486 (PMC12626828; doi:10.3389/fendo.2025.1671486)
Supplement: Supplementary file 1 [file DataSheet1.pdf]

Supplementary Appendix

Table 1. Search Strategy and Records Screened

Database	Search String	Records Retrieved	Records Screened
PubMed/ MEDLINE	(“Pheochromocytoma”[Mesh] OR pheochromocytoma* OR “Paraganglioma”[Mesh] OR paraganglioma* OR PPGL)<br>AND<br>(“Bone Neoplasms”[Mesh] OR “Bone metastases” OR “Bone metastasis” OR osseous metastases OR skeletal metastases OR “Skeletal-Related Events” OR “SRE” OR “SREs”)<br>AND<br>(“Diagnosis”[Mesh] OR diagnosis OR “Clinical presentation” OR symptoms OR “Management”[Mesh] OR treatment OR therapy)	529	302
Embase	('pheochromocytoma'/exp OR pheochromocytoma* OR 'paraganglioma'/exp OR paraganglioma* OR PPGL)<br>AND<br>('bone metastasis'/exp OR 'bone metastases' OR 'osseous metastases' OR 'skeletal metastases' OR 'skeletal-related event'/exp OR 'SRE' OR 'SREs')<br>AND<br>('diagnosis'/exp OR diagnosis OR 'clinical presentation' OR symptoms OR 'management'/exp OR treatment OR therapy)	498	199
**Total**		1027	501

Table 2. Frecquency of Bone Metastases by study

Author	Year of publication	Number of patients with mPPGL	BM Frequency. N (%)
M Schlumberger (13)	1992	20	13 (65)
Proye C et al. (14)	1992	26	6 (23.1)
Amar L et al. (15)	2007	54	37 (68.5)
Hescot S et al. (16)	2013	90	50 (56)
Ayala-Ramirez et al. (7)	2013	128	91 (71)
Abdel-Aziz TE. (17)	2015	23	13 (56.5)
Turkova H et al. (18)	2016	132	126 (95.5)
Hamidi O et al. (19)	2017	272	161 (59.1)
Yokomoto-Umakoshi M. et al (11)	2018	40	24 (60)
Ishizaki F. et al (20)	2024	15	7 (46.7)
